# Combination therapy with rituximab, low-dose cyclophosphamide, and prednisone for idiopathic membranous nephropathy: a case series

**DOI:** 10.1186/s12882-017-0459-z

**Published:** 2017-02-01

**Authors:** Frank B. Cortazar, David E. Leaf, Charles T. Owens, Karen Laliberte, William F. Pendergraft, John L. Niles

**Affiliations:** 1 Vasculitis and Glomerulonephritis Center, Division of Nephrology, Massachusetts General Hospital, 101 Merrimac St, Boston, 02114 MA USA; 2 Division of Renal Medicine, Brigham and Women’s Hospital, Boston, MA USA; 3 Division of Nephrology, Baylor University Medical Center at Dallas, Dallas, TX USA; 4 Division of Nephrology and Hypertension, Department of Medicine, University of North Carolina Kidney Center, Chapel Hill, NC USA

**Keywords:** Membranous nephropathy, Cyclophosphamide, Rituximab, Remission

## Abstract

**Background:**

Membranous nephropathy is a common cause of the nephrotic syndrome. Treatment with standard regimens fails to induce complete remission in most patients. We evaluated the efficacy of combination therapy with rituximab, low-dose, oral cyclophosphamide, and an accelerated prednisone taper (RCP) for the treatment of idiopathic membranous nephropathy.

**Methods:**

We analyzed 15 consecutive patients with idiopathic membranous nephropathy treated with RCP at Massachusetts General Hospital. Seven patients (47%) received RCP as initial therapy, and the other eight patients (53%) received RCP for relapsing or refractory disease. All patients had at least 1 year of follow-up. The co-primary outcomes were attainment of partial and complete remission. Partial remission was defined as a urinary protein to creatinine ratio (UPCR) < 3 g/g and a 50% reduction from baseline. Complete remission was defined as a UPCR < 0.3 g/g. Secondary outcomes were serious adverse events and the change in proteinuria, serum creatinine, serum albumin, cholesterol, triglycerides, and immunoglobulin G levels after 1 year of treatment.

**Results:**

Over a median follow-up time of 37 (IQR, 34–44) months, 100% of patients achieved partial remission and 93% of patients achieved complete remission at a median time of 2 and 13 months, respectively. After 1 year of treatment, median (IQR) UPCR declined from 8.2 (6.6–11.1) to 0.3 (0.2–0.7) g/g (*P* < 0.001). Three serious adverse events occurred over 51 patient years. No patients died or progressed to ESKD.

**Conclusions:**

Treatment of idiopathic membranous nephropathy with RCP resulted in high rates of complete remission. Larger studies evaluating this regimen are warranted.

**Electronic supplementary material:**

The online version of this article (doi:10.1186/s12882-017-0459-z) contains supplementary material, which is available to authorized users.

## Background

Membranous nephropathy (MN) is one of the most common causes of the nephrotic syndrome [1]. Approximately 75% of MN cases are idiopathic [2]. The course of idiopathic MN in patients not receiving immunosuppressive therapy is variable. Approximately 35% of patients achieve complete or partial spontaneous remission, while 40% of patients ultimately progress to end-stage kidney disease (ESKD) over a 10 year period [3, 4]. Risk factors for progressive renal decline, and therefore treatment with immunosuppression, include persistent proteinuria of > 4 g per day despite treatment with an angiotensin converting enzyme inhibitor (ACE-I) or angiotensin receptor blocker (ARB) and/or abnormal or worsening estimated glomerular filtration rate (eGFR) attributed to MN [5].

When immunosuppressive therapy is indicated, current guidelines recommend the Ponticelli protocol, a 6-month course of alternating monthly oral cyclophosphamide and corticosteroids [6, 7]. Alternatively, a calcineurin inhibitor-based regimen with or without low-dose prednisone is recommended if there is a desire to avoid an alkylating agent [6]. Unfortunately, these regimens generally result in rates of complete remission of < 35% [7–10].

The M-type phospholipase A2 receptor (PLA2R), a transmembrane protein expressed on glomerular podocytes, has been demonstrated to be the target antigen in most cases of idiopathic MN [11]. This creates a paradigm whereby circulating autoantibodies to PLA2R form *in situ* immune complexes at the level of the podocyte, leading to the development of MN. The central mechanistic role for autoantibodies in MN has provided a rationale for B cell targeted therapy. However, monotherapy treatment with rituximab, a monoclonal anti- CD20 antibody, fails to induce complete remission in the vast majority of patients [12–14].

In an attempt to more effectively induce remission while allowing for a reduced exposure to high-dose glucocorticoids, the practice pattern in our group for the treatment of newly diagnosed, relapsing, and refractory idiopathic MN evolved to become a three drug regimen: rituximab dosed to maintain continuous B cell depletion, a 2-month course of oral, low-dose, bridging cyclophosphamide, and an accelerated prednisone taper (RCP). Given that MN is an antibody mediated disease, the premise of this regimen is to target plasma cells with cyclophosphamide and glucocorticoids while concurrently depleting plasma cell precursors with rituximab. We present a retrospective analysis of the outcomes of 15 consecutive patients treated with this regimen.

## Methods

### Patient selection

Starting in July of 2009, combination therapy with rituximab, glucocorticoids, and cyclophosphamide became the preferred immunosuppressive regimen for patients presenting to the Massachusetts General Hospital Vasculitis and Glomerulonephritis Center with idiopathic MN. We performed a retrospective analysis of patients treated with this regimen who had biopsy-proven MN and met at least one of the following criteria: 1) persistent urinary protein to creatinine ratio (UPCR) > 4 g/g for > 6 months despite treatment with an ACE-I or ARB; 2) declining renal function, defined as a 30% rise in serum creatinine from baseline attributed to MN; 3) debilitating or life threatening symptoms due to the nephrotic syndrome; 4) recurrence of a UPCR > 4 g/g following remission after a prior immunosuppressive treatment; or 5) refractory disease, defined as failure of an alternative immunosuppressive regimen to induce a partial remission after at least 6 months of therapy. Patients were considered to have the nephrotic syndrome if they had a UPCR > 3.5 g/g, a serum albumin < 3 g/dL, and peripheral edema.

As part of routine care, all patients were evaluated for secondary causes of MN by age-appropriate cancer screening, assessment for occult malignancy as indicated based on history and physical examination, review of medications for potential drug culprits, screening for hepatitis B and C viruses, antinuclear antibody testing, and pursuing additional testing as deemed appropriate by the treating nephrologist. After retrospective chart review, patients were excluded from the analysis if they were found to have a malignancy, active hepatitis B or C, lupus membranous, or exposure to a medication known to cause MN. All patients with at least 1 year of follow-up were included in the study. The study was approved by the Massachusetts General Hospital Institutional Review Board.

### Treatment regimen

**Fig. 1:**
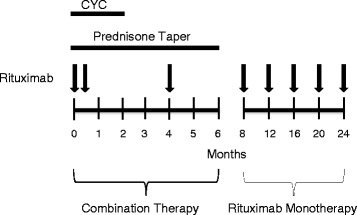
Schematic representation of treatment regimen. Cyclophosphamide was administered orally at 2.5 mg/kg daily for 1 week, then 1.5 mg/kg for daily for 7 weeks. The cyclophosphamide dose was adjusted for renal function as delineated in the methods. Prednisone was initiated at 60 mg daily, tapered to 15 mg daily by 4 weeks, and then slowly tapered to complete a 6 month course as described. Rituximab doses (*arrows*) were administered as 1000 mg intravenously. Abbreviations: CYC, cyclophosphamide

A summary of the treatment regimen is shown in Fig. 1. Cyclophosphamide was administered orally at 2.5 mg/kg daily for 1 week, then 1.5 mg/kg daily for 7 weeks. The dose of cyclophosphamide was adjusted for renal function as follows: 10% dose reduction if the eGFR was 60–90 ml/min/1.73 m^2^, 25% if the eGFR was 45–59 ml/min/1.73 m^2^, 33% if the eGFR was 30–44 ml/min/1.73 m^2^, and 40% if the eGFR was 15–29 ml/min/1.73 m^2^. GFR was estimated using the Chronic Kidney Disease Epidemiology Collaboration (CKD-EPI) equation [15]. Concurrently with the start of cyclophosphamide, patients were initiated on rituximab with two 1000 mg intravenous doses separated by approximately 2 weeks. Thereafter, patients received rituximab 1000 mg IV every 4 months for 2 years with the aim of maintaining continuous B cell depletion. The rituximab dosing interval was based on data indicating that B cell reconstitution commences 16 weeks after rituximab administration in a significant subset of patients [16]. To ensure continuous B cell depletion was achieved, peripheral blood was sent for flow cytometry analysis immediately prior to each rituximab dose. Prednisone was tapered as follows: 60 mg daily for 1 week, 40 mg daily for 1 week, 30 mg daily for 1 week, 20 mg daily for 1 week, and then a slow taper to complete a 6 month course. Minor modifications of the treatment regimen were permitted at the discretion of the treating physician.

Patients were typically seen in clinic at presentation, 1 week (first rituximab dose), 3 weeks (second rituximab dose), 2 months, 4 months, and then every 4 months during the active treatment phase. Following treatment, patients were seen every 4 to 6 months for monitoring. The following labs were checked at each visit: complete blood count (CBC), comprehensive metabolic panel, spot urine total protein, and spot urine creatinine. A CBC was checked every 2–3 weeks while patients were on cyclophosphamide to monitor for leukopenia. Lipids were checked every 4 months. Peripheral blood was sent for flow cytometry (to monitor for B cell depletion) and serum immunoglobulins prior to each rituximab infusion. Some patients were tested for antibodies to the M-type phospholipase A2 receptor (PLA2R). Testing for antibodies to PLA2R was performed on peripheral blood via enzyme-linked immunosorbent assay (Euroimmun, Lubeck, Germany) or from staining deposits on kidney biopsies for PLA2R via immunofluorescence.

### Primary outcomes

The primary outcomes were attainment of partial and complete remission. Partial remission was defined as a UPCR < 3.0 g/g and a ≥50% reduction in the UPCR from baseline. Complete remission was defined as a UPCR < 0.3 g/g. Relapse after complete or partial remission was defined as a UPCR > 3 g/g. All UPCRs were determined with a spot collection.

### Secondary outcomes

Secondary outcomes included the change in proteinuria, serum creatinine, serum albumin, total cholesterol, triglycerides, and immunoglobulin G levels from baseline to 1 year after treatment.

### Serious adverse events

At each visit, patients were asked about any interval infections, hospitalizations, or other complications. All reported adverse events were recorded in our electronic treatment flowsheets. To ensure accuracy, we report only serious adverse events, defined as those that were life threatening or required hospitalization.

### Statistical analysis

Analyses were carried out using STATA version 14 (College Station, Texas). Baseline characteristics are presented as percentages or medians and interquartile ranges (IQRs), and were compared using the Wilcoxon rank-sum test or Fisher’s exact test, as appropriate. Longitudinal differences in clinical parameters from baseline to 1 year after initiation of treatment were analyzed with the Wilcoxon signed-rank test.

The Kaplan-Meier method was used to examine the time to achieving a complete or partial remission. Time to event was the time from initiation of treatment to the attainment of complete or partial remission. The analysis was stratified by disease status (initial presentation versus relapsing/refractory disease). Differences between curves were assessed using the log-rank test and proportional-hazards model. All comparisons are two-tailed, with *P* < 0.05 considered significant.

## Results

### Baseline characteristics

In September of 2015, 15 consecutive patients with idiopathic MN treated with rituximab, cyclophosphamide, and prednisone (RCP) had at least 1 year of follow-up. Seven patients (47%) received RCP as initial therapy. The remaining eight patients (53%) were referrals from other physicians who had been previously treated for idiopathic MN and had relapsing or refractory disease. Baseline characteristics overall and stratified by treatment status are presented in Table 1.

**Table 1:** Baseline characteristics

Variable	Overall (*n* = 15)	Initial therapy (*n* = 7)	Second-line therapy (*n* = 8)	*P* value
Age (years)	62 (45–67)	52 (39–62)	64 (57–67)	0.12
Female	8 (53)	5 (71)	3 (38)	0.32
Systolic BP (mmHg)	140 (124–148)	124 (114–140)	147 (135–164)	0.037
Diastolic BP (mmHg)	82 (72–86)	80 (67–86)	83 (79–89)	0.29
Serum Creatinine (mg/dL)	1.2 (0.8–1.5)	0.8 (0.5–0.9)	1.3 (1.2–2.1)	0.011
eGFR (ml/min/1.73 m^2^)	65 (40–100)	100 (62–123)	56 (29–66)	0.024
eGFR Group				0.45
> 60 ml/min/1.73 m^2^	10 (67)	6 (86)	4 (50)	
30–60 ml/min/1.73 m^2^	3 (20)	1 (14)	2 (25)	
< 30 ml/min/1.73 m^2^	2 (13)	0 (0)	2 (25)	
UPCR (g/g)	8.2 (6.8–11.4)	6.9 (5.2–10.2)	10.1 (8.0–11.7)	0.15
UPCR group				0.41
< 4^a^	1 (7)	1 (14)	0 (0)	
4–8	5 (33)	3 (43)	2 (25)	
> 8	9 (60)	3 (43)	6 (75)	
Nephrotic Syndrome^b^	13 (87)	7 (100)	6 (75)	0.47
Albumin (g/dL)	2.7 (2.5–2.8)	2.7 (2.5–2.8)	2.6 (2.4–3.1)	0.86
Total Cholesterol (mg/dL)^c^	350 (252–371)	341 (252–367)	371 (249–438)	0.34
Triglycerides (mg/dL)^c^	172 (122–270)	134 (77–192)	177 (155–546)	0.14
Time Since Biopsy Diagnosis (months)	9 (3–24)	3 (0.5–4)	23 (16–60)	0.011
On ACE-I or ARB	13 (87)	5 (71)	8 (100)	0.20
On Statin	8 (53)	2 (29)	6 (75)	0.13
Treatment Indications				
Failure of ACE-I or ARB^d^	8 (53)	2 (29)	6 (75)	0.13
Declining Renal Function	5 (33)	1 (14)	4 (50)	0.30
Debilitating Symptoms from nephrotic syndrome	10 (67)	5 (71)	5 (63)	0.99
Relapsing Disease	6 (38)	NA	6 (75)	NA
Refractory Disease	3 (19)	NA	3 (38)	NA

Data are presented as median (interquartile range) and *n* (%)

*Abbreviations*: *ACE-I* angiotensin converting enzyme, *ARB* angiotensin receptor blocker, *BP* blood pressure, *eGFR* estimated glomerular filtration rate, *UPCR* urinary protein: creatinine ratio

^a^The patient with a UPCR < 4 g/g had decompensated nephrotic syndrome with refractory edema and an albumin nadir of 1.1 g/dL

^b^Nephrotic syndrome was defined as urinary protein: creatinine ratio > 3.5 g/g, serum albumin < 3 g/dL, and peripheral edema

^c^One patient in the second-line therapy group did not have baseline cholesterol or triglycerides

^d^Failure of ACE-I or ARB was defined as a persistent UPCR > 4 g/g despite at least 6 months of therapy

The median age at presentation was 62 years (IQR, 45–67 years) and 53% of patients were female. The study population consisted of 13 Caucasian patients, 1 black patient, and 1 Hispanic patient. Baseline autoantibodies to PLA2R were present in 4 of 5 patients with available data. The most common treatment indication for patients receiving RCP as initial therapy was debilitating symptoms of the nephrotic syndrome. Patients receiving RCP as second-line therapy were most commonly treated for a disease relapse (UPCR > 4 g/g) occurring after prior disease remission induced by immunosuppression and failure of 6 months of an ACE-I or ARB to result in a reduction of UPCR to < 4 g/g. All patients receiving RCP as second-line therapy and 1 patient receiving RCP as initial therapy met more than 1 treatment indication.

The median UPCR was 8.2 (IQR, 6.8–11.4) g/g and was similar in patients receiving RCP as initial versus second-line therapy. The majority of patients in both treatment groups had the nephrotic syndrome. Baseline serum creatinine was higher in patients receiving RCP as second-line therapy compared to initial therapy (median [IQR], 1.3 [1.2–2.1] versus 0.8 [0.5–0.09] mg/dl, *P* = 0.011). Three patients had an eGFR 30–60 ml/min/1.73 m^2^, and two patients had an eGFR < 30 ml/min/1.73 m^2^.

Of the 8 patients who received prior treatment, 4 (50%) had been treated with 2 different treatment regimens. The 8 patients with relapsing or refractory disease were exposed to 12 different treatment regimens. In combination with prednisone, 3 patients were treated with cyclophosphamide, 2 with mycophenolate mofetil, 3 with cyclosporine, and 1 with tacrolimus. Three patients received prednisone monotherapy.

The five patients treated with RCP for disease relapse had a median immunosuppression-free interval of 18 months (IQR, 12 to 84 months). In all cases, the UPCR at initiation of RCP was greater than the UPCR 6 months prior (median difference 1.5 g/g [IQR, 0.6 to 4.1 g/g]). The characteristics of patients treated for refractory disease are presented in Table 2.

**Table 2:** Characteristics of patients with refractory disease

Patient	Regimen	Duration of therapy (mos)	UPCR 6 months before RCP	UPCR at RCP initiation
1	MMF & Pred	12	5.2	10.7
2	CsA & Pred	18	NA	11.9
3	CsA & Pred	6	6.8	16.7

*Abbreviations*: *CsA* cyclosporine, *MMF* mycophenolate mofetil, *mos* months, *NA* not available, *Pred* prednisone, *UPCR* urinary protein: creatinine ratio, *RCP* rituximab, cyclophosphamide, prednisone

### Primary outcomes

**Fig. 2:**
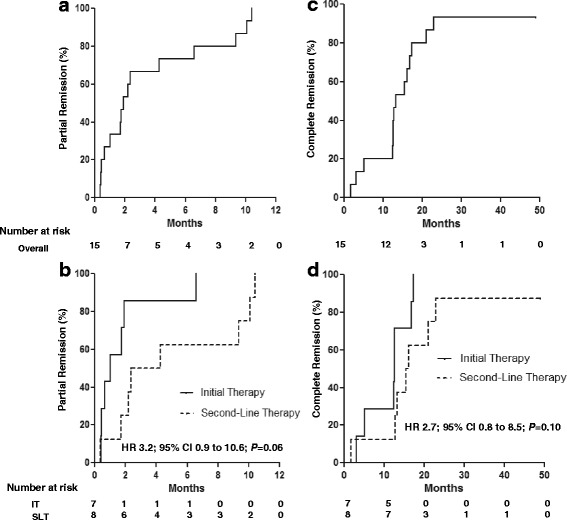
Kaplan-Meier curves for partial and complete remission. Kaplan-Meier curves for the overall group and stratified by treatment status are shown for partial remission (panels **a** and **b**) and complete remission (panels **c** and **d**). Abbreviations: HR, hazard ratio; IT, initial therapy; SLT, second-line therapy

Median follow-up time was 37 (IQR, 34–44) months. All patients achieved partial remission by 1 year. Median time to achievement of partial remission was 2 (IQR, 1–7) months (Fig. 2a). There was a trend toward faster induction of partial remission in patients receiving RCP as initial treatment versus second-line therapy (HR 3.2; 95% CI 0.9–10.6; *P* = 0.06, Fig. 2b).

Fourteen patients (93%) achieved complete remission at a median time of 13 (IQR, 12–17) months (Fig. 2c). All 14 patients who achieved complete remission had a subsequent confirmatory UPCR < 0.3 g/g (Additional file 1: Figure S1). The one patient who failed to achieve complete remission had advanced CKD attributed to MN with an eGFR of 18 ml/min/1.73 m^2^ at initiation of RCP. Like with attainment of partial remission, there was a trend to more rapid induction of complete remission in patients receiving RCP as initial therapy (HR 2.7; 95% CI 0.8–8.5; *P* = 0.10, Fig. 2d). Antibodies to PLA2R became undetectable after treatment with RCP in the 3 patients with documented circulating antibodies at baseline

Additional treatment was required in 2 patients who had persistent nephrotic-range proteinuria at their 4 month follow-up visit. Both of these patients received an additional 2 month course of cyclophosphamide at 1 mg/kg and subsequently achieved complete remission. One patient relapsed 9 months after partial remission while B cells remained depleted, but achieved complete remission after treatment with an additional 1 month of cyclophosphamide at 1.5 mg/kg.

At last follow-up the full 2-year regimen was completed by 14 patients, 8 of whom had relapsing or refractory disease. Following completion of the 2-year regimen, one relapse occurred over 20 patient-years of treatment-free follow-up (median treatment-free follow-up, 13.7 [IQR, 10 to 19] months). The relapse occurred 4.8 years after the patient’s last rituximab dose. At the time of relapse, B cells had reconstituted and accounted for 16.7% of the lymphocyte pool. This patient was treated with an 8 week course of cyclophosphamide at 1.5 mg/kg, a rapid prednisone taper, and two doses of rituximab 1000 mg IV separated by 2 weeks. The patient was back in complete by remission by 6 months.

### Secondary outcomes

**Fig. 3:**
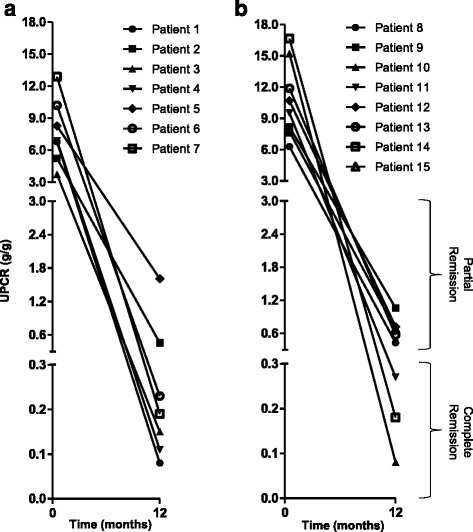
Change in proteinuria after 1 year of treatment. Data are depicted for patients receiving RCP as initial therapy (panel **a**) or as second-line therapy (panel **b**). Abbreviations: UPCR, urinary protein:creatinine ratio

Response to treatment with RCP at 1 year, both overall and stratified by treatment status, is shown in Table 3. UPCR fell from a median of 8.2 (IQR, 6.6–11.1) to 0.3 (IQR, 0.2–0.7) g/g (*P* < 0.001, Fig. 3).

**Table 3:** Response to treatment at 1 year

	Overall (*n* = 15)			Initial therapy (*n* = 7)			Second-line therapy (*n* = 8)		
Variable	Baseline	1-Year	*P* value	Baseline	1-Year	*P* value	Baseline	1-Year	*P* value
UPCR (g/g)	8.2 (6.6–11.1)	0.3 (0.2–0.7)	<0.001	6.9 (5.2–10.2)	0.2 (0.1–0.5)	0.02	10.1 (8–11.7)	0.6 (0.3–0.7)	0.01
Creatinine (mg/dL)	1.2 (0.8–1.5)	1.0 (0.8–1.2)	0.44	0.8 (0.5–0.9)	0.8 (0.7–1.0)	0.13	1.3 (1.2–2.1)	1.2 (1.1–1.7)	0.02
Albumin (mg/dL)	2.7 (2.5–2.8)	4.2 (4–4.4)	<0.001	2.7 (2.5–2.8)	4.2 (3.9–4.4)	0.02	2.6 (2.4–3.1)	4.2 (4.0–4.4)	0.01
Cholesterol (mg/dL)^a^	350 (252–371)	189 (148–220)	0.001	341 (252–367)	167 (128–191)	0.02	371 (249–438)	207 (155–251)	0.02
Triglycerides (mg/dL)	172 (122–270)	189 (148–220)	0.40	134 (77–192)	105 (80–244)	0.74	177 (155–546)	182 (152–204)	0.24
IgG (mg/dL)^b^	314 (199–551)	730 (538–908)	0.003	499 (296–731)	806 (676–1027)	0.02	199 (198–308)	611 (364–676)	0.08

Data are presented as median (interquartile range). Baseline and 1 year values compared with Wilcoxon signed-rank test

^a^1 patient in the second-line therapy group did not have baseline cholesterol or triglycerides

^b^three patients in the second-line therapy group had missing baseline IgG

*Abbreviations*: *IgG* immunoglobulin G, *UPCR* urinary protein:creatinine ratio

Overall, there was no change in serum creatinine at 1 year. However, in patients receiving RCP as second-line therapy, there was a significant fall in serum creatinine (1.3 [IQR, 1.2–2.1] to 1.2 [IQR, 1.1 to 1.7] mg/dL; *P* = 0.02). There was a significant rise in serum albumin and a significant decline in cholesterol in both treatment groups (Table 3). Despite treatment with rituximab, there was a significant rise in IgG levels (314 [IQR, 199–551] to 730 [IQR, 538–908] mg/dL; *P* = 0.003) due to a reduction in urinary losses.

Ten patients had at least 1 year of follow-up after completion of the 2-year RCP regimen. There was no difference in UPCR or serum creatinine 1 year after treatment completion compared with 1 year after treatment initiation (Table 4).

**Table 4:** Response to treatment at 3 years

Variable	1 year after RCP initiation (*n* = 10)	1 year after RCP completion (*n* = 10)	*P* value
UPCR (g/g)	0.3 (0.2–0.7)	0.1 (0.1–0.2)	0.09
Albumin (mg/dL)	4.2 (4.0–4.4)	4.6 (4.3–4.7)	0.036
Creatinine (mg/dL)	1.0 (0.8–1.2)	1.1 (0.8–1.3)	0.11

Data are presented as median (interquartile range) for the 10 patients with 1 year of treatment-free follow-up. Values 1 year after RCP initiation were compared to values 1 year after RCP completion with the Wilcoxon signed-rank test

*Abbreviations*: *UPCR* urinary protein:creatinine ratio, *RCP* rituximab, cyclophosphamide, prednisone

### Serious adverse events

Over a combined follow-up time of 51 patient years, three serious adverse events (SAE) s occurred. Two SAEs, an episode of late-onset neutropenia and a febrile viral syndrome, were felt to be potentially treatment-related. Both events occurred in patients receiving RCP as second-line therapy. The episode of late-onset neutropenia occurred 3.7 months after the patient’s 6^th^ rituximab dose and promptly responded to treatment with filgrastim. The patient subsequently received two additional doses of rituximab without incident. The patient with a viral syndrome was hospitalized for evaluation of fever and malaise. An infectious workup, including blood and urine cultures, was negative and the symptoms resolved with only supportive care. A specific virus was not isolated, but the course was most consistent with a viral process.

The third SAE was an episode of altered mental status that occurred after the initiation of high-dose gabapentin. The patient’s mental status returned to baseline following withdrawal of gabapentin, and this SAE was not felt to be a consequence of MN or the RCP regimen. No patients died or progressed to ESKD.

## Discussion

Treatment with RCP was effective at inducing remission in patients with idiopathic MN. In patients treated for at least 1 year, 100% achieved partial remission and 93% achieved complete remission. The response was durable, with only 1 patient sustaining a relapse after completion of treatment. Furthermore, achieving complete remission appears to be time-dependent, with most individuals achieving complete remission greater than 1 year after initiating therapy. However, treatment with a standard regimen of an alkylating agent and glucocorticoids generally achieves complete remission in less than 35% of patients, even after extended follow up [3, 7, 9]. Similarly, in a report of 100 patients with MN treated with rituximab monotherapy, complete remission occurred in fewer than 30% of patients [14].

Despite the limited sample size, our results suggest that rates of remission achieved with RCP may be superior to other treatment regimens that have been reported. We attribute the success of the regimen to a multi-targeted approach to halt the production of pathogenic antibodies causing MN. Specifically, prednisone and cyclophosphamide target plasmablasts and plasma cells, while rituximab depletes the B cell pool required to replete the plasma cell population. By administering rituximab every 4 months, B cells are prevented from reconstituting. Standard cytotoxic regimens for MN can be circumvented if plasmablasts and plasma cells producing pathogenic antibody are restored from the B cell population. Likewise, rituximab monotherapy is unlikely to be effective if long-lived plasma cells are participating in the disease process.

We hypothesize that the long duration of B cell depletion with RCP will lead to a more sustained remission compared with alternative regimens. Following completion of the RCP regimen, 1 relapse occurred over 20 patient-years in a population enriched in patients with relapsing and refractory disease. Prior studies using cytotoxic therapy with prednisone have resulted in relapse rates of approximately 30% by 1.5 years, and the relapse rate approaches 45% for calcineurin inhibitor-based regimens [7, 8]. More data and longer follow-up are needed to more accurately define the relapse rate associated with RCP. The optimal treatment regimen for patients with relapsing disease following RCP is unknown. The one patient who sustained a relapse after a treatment-free interval of 4.8 years was successfully retreated with the RCP regimen. It is possible that early and non-severe relapses may respond well to rituximab alone.

The high rate of complete remission in patients treated with RCP is likely to lead to long-term clinical benefit. Patients attaining complete remission have an excellent renal prognosis and a significantly lower rate of reaching ESKD than those who achieve only partial remission [17, 18]. In one analysis of 348 MN patients with a median follow-up time of 6 years, none of the 102 patients with complete remission progressed to stage V CKD, whereas 9% of the 132 patients with partial remission progressed to stage V CKD [17]. Likewise, the slope of renal decline was significantly flatter in patients who achieved complete remission [17]. Additionally, since albuminuria is a strong independent risk factor for coronary artery disease and stroke, maximizing the reduction of proteinuria in patients with MN with RCP may have important extra-renal benefits as well [19].

There may be concern that the simultaneous use of 3 immunosuppressive medications will lead to an increased risk of infectious complications and other adverse events. In our study, three serious adverse events occurred over 51 patient years. This regimen allows for a rapid prednisone taper in which patients are reduced to a dose of 15 mg per day by 4 weeks. Moreover, the cumulative dose of cyclophosphamide used in this regimen (97 mg/kg prior to renal adjustment) is significantly less than in other regimens for MN. For example, in the Ponticelli regimen, patients receive a total of 180 mg/kg of cyclophosphamide. Adjustment of the cyclophosphamide dose for renal function likely serves to further mitigate potential side effects. The risk of urothelial carcinoma is dose-dependent, and is unlikely to occur with the doses used in our regimen [20, 21]. Cyclophosphamide has also been associated with the development of myelodysplastic syndrome, leukemia, and other malignancies in a dose-dependent fashion [22, 23] . However, cyclophosphamide in the RCP regimen is used for a shorter duration and at a lower dose than conventional regimens used for the treatment of renal or rheumatologic diseases. Thus, while it is difficult to estimate the exact risk of malignancy with RCP, it is likely lower than the incidence associated with other cyclophosphamide-based regimens.

Rituximab in our protocol was dosed every 4 months for 2 years with the aim of maintaining continuous B cell depletion. This represents a longer duration of therapy than other regimens using rituximab for idiopathic MN [12–14, 24]. Our rationale was that more prolonged therapy would lead to a higher rate of complete remission and lead to lower rates of relapse without a significant increase in adverse events. Indeed, achieving complete remission appeared to be time-dependent, with most individuals achieving complete remission greater than 1 year after initiating therapy. Moreover, the response appeared durable with only 1 patient sustaining a relapse after complete remission was attained. A prior investigation of 173 patients with ANCA vasculitis demonstrated that rituximab-induced continuous B cell depletion for a median duration of 2.1 years was safe and effective with survival that mirrored that of the general population [25]. While the RCP regimen was well tolerated during the observation-period, we cannot exclude the possibility of treatment-related side effects developing in the future.

Our study has several important limitations. Patient selection in studies of MN is important to consider given the possibility of spontaneous remission in low-risk patients [2]. Inclusion of patients with a higher probability of spontaneous remission can result in overestimation of treatment efficacy. Of note, our study population was enriched in women compared to the male predominance typically observed in MN. There is evidence that female patients may have a better prognosis and a higher rate of spontaneous remission [26, 27]. In addition, some patients receiving RCP as initial therapy were treated early in their disease course due to debilitating nephrosis, and we cannot exclude the possibility that a subset would have remitted spontaneously. However, 50% of the patients in our study had relapsing or refractory disease, and the high-grade baseline proteinuria (median UPRC of 8.2 g/g) decreases the probability of spontaneous remission [28]. Moreover, the rate of spontaneous complete remission in idiopathic MN is low. In an observational study of 100 patients with predominantly low-risk disease, only approximately 10% of patients achieved complete remission by 2 years [29]. Thus, it is unlikely that the high rates of remission that we observed with RCP can be attributed to spontaneous remissions.

The discovery of antibodies to PLA2R as the inciting event in the majority of cases of idiopathic MN has greatly advanced the mechanistic understanding of the disease [11]. In addition, a decline in anti-PLA2R antibodies following treatment with rituximab predicts, and typically precedes, clinical response [30]. Although treatment with RCP led to undetectable PLA2R antibodies in the three patients with documented baseline circulating antibodies, our limited data precluded a robust analysis of clinical response with a change in antibody titers.

An additional limitation is that a spot UPCR, rather than a 24-h urine collection, was used to quantify proteinuria. This study is a retrospective analysis of patients undergoing routine clinical care, a setting in which the 24-h urine collection is cumbersome and often collected inappropriately. The UPCR has excellent correlation with 24-h urine collection [31]. However, the UPCR is subject to more intra-person variability and is influenced by the rate of urinary creatinine excretion [32]. Nonetheless, all patients who achieved complete remission had multiple subsequent UPCR values of < 0.3 g/g after attaining complete remission. Given the repeated confirmation of complete remission in our patients, it is unlikely that the variability of the UPCR significantly influenced the results.

Finally, the study is single-center, of limited size, and lacks a comparator group. Statistical results must be interpreted with caution in this setting. Larger studies are required to confirm our findings.

## Conclusions

In summary, RCP was effective and safe for the treatment of idiopathic MN and may allow for a remission rate superior to existing regimens. Over a median follow-up time of 37 months, 100% of patients achieved partial remission and 93% of patients achieved complete remission. In addition, outcomes were similar regardless of incident or relapsing disease. Larger studies comparing RCP to established treatments are needed to further assess the utility of this regimen in the treatment of idiopathic MN.

## Additional file

Additional file 1: Figure S1. Change in proteinuria with treatment. Data are depicted for patients receiving RCP as initial therapy (panel A) or as second-line therapy (panel B). One patient (Patient 9 in panel B) did not achieve complete remission. After achieving complete remission, all patients had a subsequent urinary protein:creatinine ratio < 0.3 g/g. Abbreviations: UPCR, urinary protein:creatinine ratio. (DOCX 55 kb)

## Abbreviations

ACE-I: Angiotensin converting enzyme inhibitor
ARB: Angiotensin receptor blocker
eGFR: Estimated glomerular filtration rate
ESKD: End-stage kidney disease
MN: Membranous nephropathy
PLA2R: Phospholipase A2 receptor
RCP: Combination rituximab, low-dose oral cyclophosphamide, and accelerated prednisone taper
UPCR: Urinary protein to creatinine ratio
