# Supplementary material for: Combination therapy with rituximab, low-dose cyclophosphamide, and prednisone for idiopathic membranous nephropathy: a case series
Source: BMC Nephrol. 2017 Feb 1;18:44. doi: 10.1186/s12882-017-0459-z (PMC5286562; doi:10.1186/s12882-017-0459-z)
Supplement: Additional file 1: Figure S1. — Change in proteinuria with treatment. Data are depicted for patients receiving RCP as initial therapy (panel A) or as second-line therapy (panel B). One patient (Patient 9 in panel B) did not achieve complete remission. After achieving complete remission, all patients had a subsequent urinary protein:creatinine ratio < 0.3 g/g. Abbreviations: UPCR, urinary protein:creatinine ratio. (DOCX 55 kb) [file 12882_2017_459_MOESM1_ESM.docx]

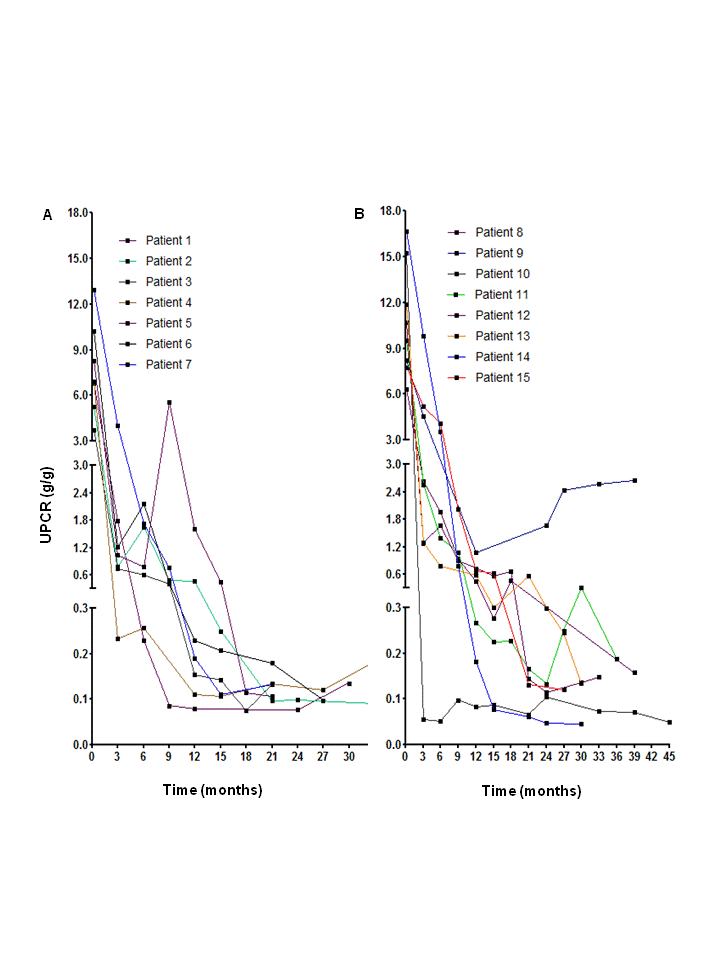


**Additional file 1: Figure S1.** Change in proteinuria with treatment. Data are depicted for patients receiving RCP as initial therapy (panel A) or as second-line therapy (panel B). One patient (Patient 9 in panel B) did not achieve complete remission. After achieving complete remission, all patients had a subsequent urinary protein:creatinine ratio < 0.3 g/g. Abbreviations: UPCR, urinary protein:creatinine ratio.
